# RunicNet: Leveraging CNNs With Attention Mechanisms for Cervical Cancer Cell Classification

**DOI:** 10.1177/11795972251351815

**Published:** 2025-07-17

**Authors:** Erin Beate Bjørkeli, Morteza Esmaeili

**Affiliations:** 1 Department of Diagnostic Imaging, Akershus University Hospital, Lørenskog, Norway; 2 Institute of Clinical Medicine, University of Oslo, Norway; 3 Department of Electrical Engineering and Computer Science, University of Stavanger, Norway

**Keywords:** cancer screening, cell classification, deep learning, early cancer detection, pap smear, pixel attention

## Abstract

**Introduction:**

Early detection through routine screening methods, such as the Papanicolaou (Pap) test, is crucial for reducing cervical cancer mortality. However, the Pap smear method faces challenges including subjective interpretation, significant variability in diagnostic confidence, and high susceptibility to human errors—leading to both false negatives (missed abnormalities) and false positives (unnecessary follow-up procedures). Providing a first opinion could improve the screening examination pipeline and greatly aid the specialist’s confidence in reporting. Artificial intelligence (AI)-based approaches have shown promise in automating cell classification, reducing human error, and identifying subtle abnormalities that may be missed by experts.

**Methods:**

In this study, we present RunicNet, a CNN-based architecture with attention mechanisms designed to classify Pap smear cell images. RunicNet integrates attention mechanisms such as High-Frequency Attention Blocks-enhanced Residual Blocks for improved feature extraction, Pixel Attention for computational efficiency, and a Gated-Dconv Feed-Forward Network to refine image representation. The model was trained on a dataset of 85 080 cell images, employing data augmentation and class balancing techniques to address dataset imbalances.

**Results:**

Evaluated on a separate testing dataset, RunicNet achieved a weighted F1-score of 0.78, significantly outperforming baseline models such as ResNet-18 (F1-score of 0.53) and a fully connected CNN (F1-score of 0.66).

**Discussion:**

The findings support the potential of attention-based CNN models like RunicNet to significantly improve the accuracy and efficiency of cervical cancer screening. Integrating such AI systems into clinical workflows may enhance early detection and reduce diagnostic variability in Pap smear analysis.

## Introduction

Cervical cancer was responsible for an estimated 340 000 deaths among women worldwide in 2020.^1^ Developing countries account for 80% of these deaths, largely due to inadequate medical infrastructure and a shortage of qualified pathologists.^2^ In contrast, the widespread use of the Papanicolaou (Pap) smear in developed nations has significantly reduced cervical cancer mortality by facilitating early detection.^3,4^ Underdeveloped regions continue to struggle with providing accessible and affordable care.

Despite its success in developed nations, the Pap smear method, which relies on manual microscopic examination of cell samples by pathologists or cytotechnologists,^4^ has several notable limitations. These limitations, including moderate sensitivity, the potential for false-positive outcomes, and a typical turnaround time of more than a week,^5^ highlight the need for more efficient and accurate diagnostic approaches. False-positive results may incorrectly suggest the presence of abnormal or cancerous cells, leading to unnecessary follow-up tests and causing anxiety for patients.^6^ Hence, developing an automated tool for analyzing Pap smear cell images can potentially accelerate reporting, improve accuracy and specificity, and enhance confidence in diagnostics. Providing a first-opinion system could streamline the screening pipeline and greatly aid specialists in their assessments.

Artificial intelligence (AI) has shown promise in automating Pap smear analysis, with the potential to address the limitations of manual screening.^7^ AI can detect subtle abnormalities that may be missed by experts (improving sensitivity), reduce false negatives (improving accuracy), and facilitate a cost-effective diagnostic approach.^7,8^ As a subcategory of AI, convolutional neural network (CNN) approaches have been widely applied to classification tasks within the medical imaging domain.^9^ Even though CNNs have demonstrated some success in cervical cancer image classification, with studies focusing on detecting abnormal Pap smear cells using standard feedforward networks or employing classical machine learning (ML) methods,^10^ and others utilizing CNN models to classify cervical cell images,^11-14^ these architectures can face challenges in effectively capturing long-range contextual information and may be susceptible to overfitting, particularly with limited datasets. Chitra and Kumar^11^ applied a CNN-long short-term memory classifier, achieving greater than 90% accuracy, outperforming classical ML classifiers. Araújo et al^12^ applied CNNs to segment unhealthy cells acquired by manually identified regions of interest from microscopy, and Liu et al^13^ implemented a similar approach to classify unhealthy cells using a Pulse CNN with an improved ResNet-50 trained on cervical cell images, which were carefully prepared by manual extraction of microscopic images. However, CNNs are limited in their ability to capture long-range dependencies and contextual relationships within the data, especially when confronted with variations in cell structures across images.^10,15^

Addressing the limitations of conventional CNNs—such as their tendency to overfit on small datasets^16^—recent advances in attention mechanisms offer a promising solution. Attention modules can enhance a model’s ability to focus on relevant features and capture long-range dependencies, leading to improved performance in tasks like image classification and medical diagnosis.^17,18^ In this study, we introduce RunicNet, a CNN-based model enhanced with efficient attention mechanisms to improve the accuracy and robustness of cervical cancer cell classification. Specifically, RunicNet integrates High-Frequency Attention Blocks^19^ and Pixel Attention,^20^ which allow the model to better capture fine-grained and edge-sensitive features crucial for Pap smear image analysis. These components contribute to more precise diagnostic predictions, supporting early detection and improved patient outcomes.

## Materials and Methods

### Dataset

We acquired Pap smear cell data from an open-source repository, consisting of separate training and testing datasets.^21^ The dataset includes annotated images from cervical cancer screenings, in which cell images were extracted in 2021. The authors began the screening process with a Pap smear test, where cell samples were collected from the cervix and prepared as smears on glass slides. These slides were then digitized using high-resolution scanners, allowing for the extraction and classification of individual cells. Analysis of these datasets began with identifying regions of interest within the smear to locate relevant cells. The smears were then divided into smaller sections for efficient processing. Advanced segmentation algorithms were applied to isolate individual cells, removing background artifacts and ensuring the focus remains on critical cellular structures essential for accurate classification.

**Figure 1.**
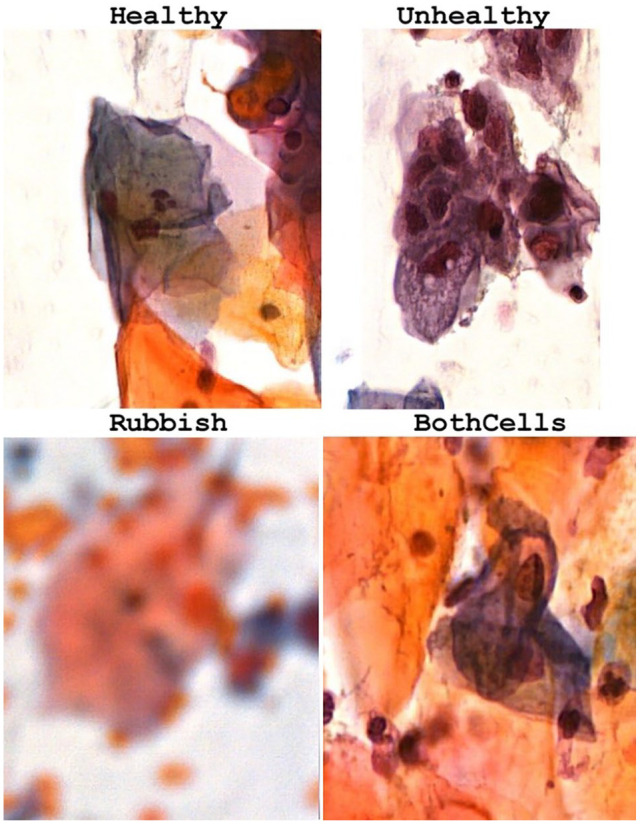
Example images of the 4 categories used for model training.

The dataset classification framework organizes cervical cells into 4 distinct categories: “healthy” (normal cytology), “unhealthy” (indicating precancerous or cancerous changes), “rubbish” (poor-quality images unsuitable for diagnosis), and “bothcells” (composite images containing both healthy and unhealthy cells; Figure 1 and Table 1). The inclusion of the “bothcells” category addresses the complexity of real-world samples, enhancing the validity and diagnostic applicability of classification systems. To evaluate model performance, the test dataset comprises unlabeled images from 3 classes: healthy, unhealthy, and rubbish, providing a robust benchmark for assessing classification accuracy and generalizability. In this study, and because the “bothcells” class was removed from the test dataset obtained from,^21^ we treated “bothcells” as an “unhealthy” class when evaluating the model’s performance (Table 1).

**Table 1.** The number of images used for training and testing of the deep learning models.

Dataset	Healthy	Unhealthy	Rubbish	Bothcells	Total
Training set	28 895	1930	50 371	3884	85 080
Testing set	5826	1012	11 757	0	18 595

### Model Architecture

**Figure 2.**
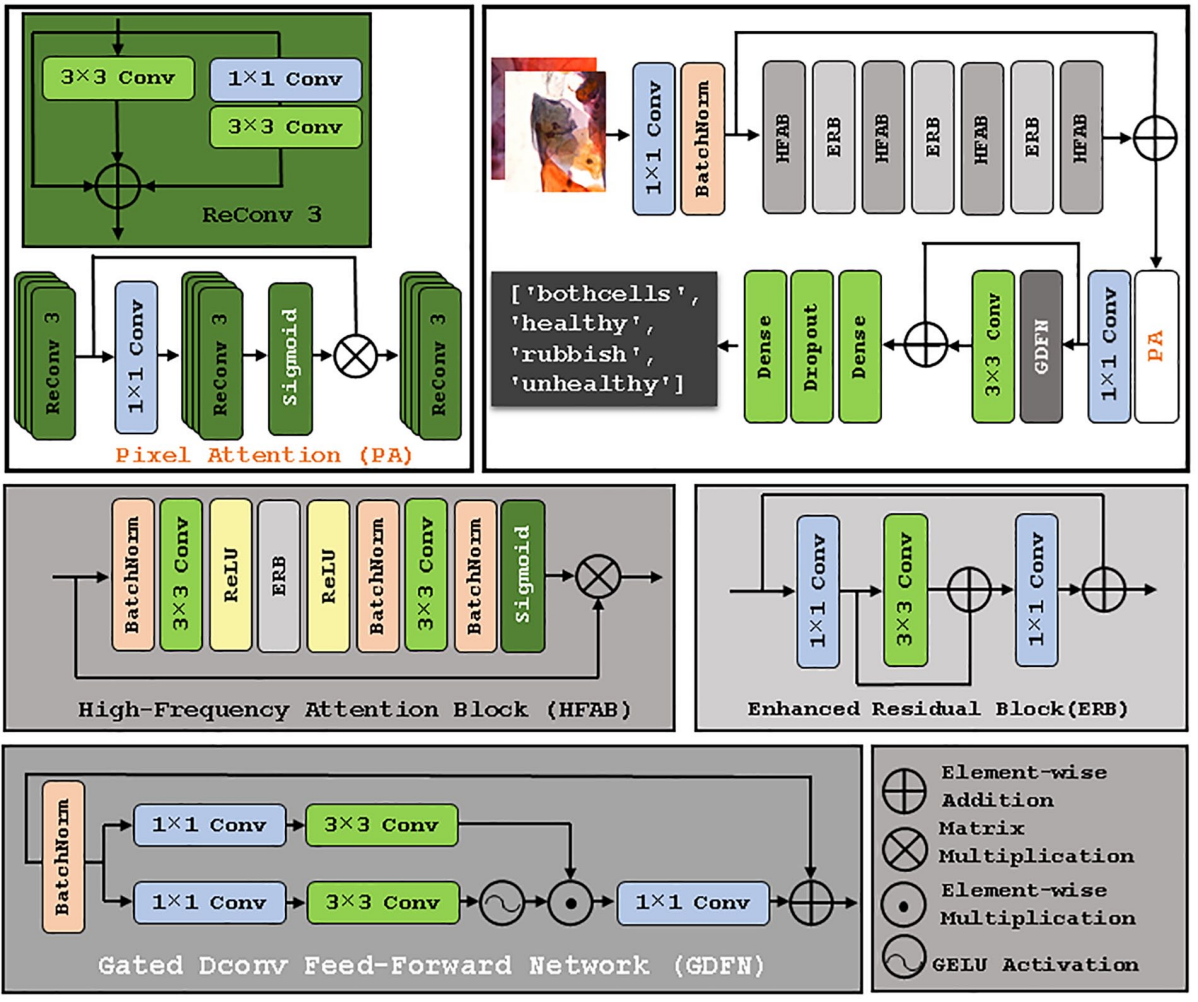
RunicNet architecture. Deep learning model architecture includes specialized convolutional and attention-based blocks: Re-parameterized 3 × 3 Convolution (Re Conv3) and Pixel Attention (PA) to improve model’s efficiency mechanism and feature generation; High-Frequency Attention Block (HFAB) which maintains sharpness and fine details; Enhanced Residual Block (ERB) which preserves edges and textures; and Gated Dconv Feed-Forward Network (GDFN) which selectively filters important features.

The proposed RunicNet model consists of a sequential arrangement of Enhanced Residual Blocks (ERB), High-Frequency Attention Blocks (HFAB),^19^ Pixel Attention (PA),^20^ and a Gated Dconv Feed-Forward Network (GDFN) as a transition block,^22^ with the initial number of feature maps set to 64. Following the HFAB stack is the PA block forming a sequential architectural pipeline. The input to the model is 196 × 196 × 3 RGB cell images, passed through an initial 1 × 1 convolutional layer with 64 filters, stride 1, and padding “same,” followed by Batch Normalization. The feature extractor includes stacked (HFAB→ ERBs) × 3 → HFAB → PA block → GDFN. The GDFN block selectively filters important features and serves as the transition layer. At the end of the network body, a global average layer, dense layer, and dropout layer (dropout rate of 20%) are included to aggregate final features and prevent overfitting. The model operates with 64 channels and produces 4 output channels. Figure 2 illustrates the arrangement of these layers within the model.

The model architecture is structurally similar to standard feedforward neural networks; however, RunicNet incorporates additional operations and skip connections that enhance its effectiveness in feature extraction tasks. HFAB-ERB pairs are positioned first to collectively extract low- and mid-level spatial features while preserving information through residual learning and emphasizing edge and boundary details. HFAB is an innovative mechanism designed to improve CNN performance by focusing on high-frequency regions, such as edges while maintaining computational efficiency.^19^ HFAB utilizes a streamlined sequential attention branch with efficient 3 × 3 convolutions, and adjusts each position based on its neighboring pixels, targeting high-frequency areas crucial for image classification.^19^ Specifically, HFAB applies a high-pass filter via 3 × 3 conv to enhance edge and texture information. To further optimize performance, Batch Normalization is integrated into the attention branch, stabilizing training and ensuring feature values remain within the Sigmoid effective range.

ERB contains two 3 × 3 convolutional layers (64 filters), each followed by Batch Normalization and ReLU activation. A skip connection adds the block input to the output of the second convolutional layer, allowing gradient flow and preserving spatial information. Down-sampling (via stride or pooling) is applied where needed to reduce feature map dimensions.

Pixel attention^20^ was incorporated into the model architecture before the transition block to enhance feature generation and improve network efficiency. PA follows to refine pixel-level attention after frequency enhancement. As illustrated in Figure 2, two additional branches were introduced alongside the original 3 × 3 convolution. These branches consist of an identity shortcut and 2 cascaded convolution layers of sizes conv 1 × 1 and conv 3 × 3. This small block provides a re-parameterized block (ReConv 3 × 3).^23,24^ Since the operations of the 3 branches are completely linear, the re-parameterized backbone can be converted to a single conv 3 × 3 for inference. The outputs from the 3 branches are summed before the activation layer. The PA block consists of a stack of ReConv 3 × 3 followed by a conv 1 × 1, a stack of ReConv, a Sigmoid function, and ReConv 3 × 3 that generates a 3-dimensional attention coefficient map.

This coefficient map is applied multiplicatively to the input features to guide attention at the pixel level. The use of re-parameterized convolutions allows the network to operate efficiently during training and compress down to a lightweight backbone for inference.

Gated-Dconv Feed-Forward Network block (Figure 2) was used as a feed-forward network transformer model,^22^ including 2 fully connected layers with a non-linearity in between, Gaussian Error Linear Units (GELU).^25^ This modified transformation layer has a gating mechanism to enhance information flow through the network.^22^ The gating layer follows an element-wise product of 2 linear projection layers, one of which is activated using the GELU non-linearity. Using GELU enables equal attention in a spatial context, improving the model’s attention to local image structures. GDFN regulates the propagation of complementary features, enabling subsequent network layers to concentrate on more refined image attributes.^22^ This selective approach enhances the quality of the final output. In our implementation, GDFN includes a depth-wise separable convolution applied to the output of the element-wise gated multiplication, enabling efficient spatial filtering.

To evaluate and compare the performance of RunicNet with traditional CNN architectures, we implemented 2 CNN-based models: ResNet-18 and the fully connected (FC) CNN with 8 layers. These models were trained and tested under similar conditions to those of RunicNet, and their performance was compared based on accuracy, precision, and loss on the validation dataset. This comparison provides insight into the effectiveness of the RunicNet relative to conventional CNN architectures.

### Implementation Details

The implementation of the RunicNet network utilized a total of 85 080 images for training, which were split into 59 556 images (70%) for training and 25 524 images (30%) for validation. For training, patches of size 196 × 196 were cropped from the original images. Due to class imbalance, where the “unhealthy” class had significantly fewer samples, we augmented unhealthy images using random flipping and rotation to enhance generalization. Additionally, class weights were assigned during training to prevent bias toward the majority class.^26^ The adjustable weighting option is available in Keras and TensorFlow, increasing the importance of the minority class and enabling generalization.^27,28^ In out implementation, we assigned the “unhealthy” a weight five times greater than other classes during training. To further mitigate overfitting and enhance model generalization, we incorporated L2 regularization (weight decay of 0.001) into the convolutional layers.

The model was developed using TensorFlow 2.5^27^ and Keras,^28^ with a total of 2.59 M trainable parameters. For model optimization, we used categorical cross-entropy loss (equation (1)) as the objective function. This loss function is suitable for multi-class classification tasks, as it effectively penalizes incorrect high-confidence predictions while encouraging the model to output probability distributions that align with the true class labels.^29^ The loss function was minimized using the Adam optimizer.^30^ The network was trained over 49 epochs with a batch size of 64, and the performance was evaluated and monitored based on accuracy, precision, and loss on the validation set (30% of the training dataset). Using various modifications—such as optimized layer arrangements, number of loops for ReConv 3 × 3, and hyperparameter tuning (eg, exploring learning rates from 10^−6^ to 10^−4^)— we implemented further refining the models’ classification accuracy and robustness. The initial learning rate was set at 10⁻⁵, which is halved every 25 iterations. The training was performed on a system with an NVIDIA A100 40 GB GPU and 94 GB of RAM, where approximately 3 GB of memory was utilized during training.

(1) $$logloss=-\frac{1}{N}\overset{N}{\underset{i=1}{\sum }}\overset{M}{\underset{j=1}{\sum }}y_{ij}\log\left(p_{ij}\right)$$

where *N* denotes the number of images in the test set, *M* is the number of classes, *y_ij_* is the ground truth label indicating that image *i* belongs to class *j*, and *p_ij_* is the predicted probability of image *i* belonging to class *j*.

The ResNet-18 model was constructed using the ResNet-18 architecture from TensorFlow/Keras Applications, with the top FC layers removed to allow for customization. The final FC layer was replaced with a Dense layer, consisting of 4 output units corresponding to the number of classes. The base model was frozen to retain the pre-trained weights from ImageNet, preventing modification during training. The custom layers on top were fine-tuned to learn task-specific features. The total number of parameters in the model was 25.69 M, with 2.10 M trainable parameters corresponding to the newly added layers. The model was compiled using the Adam optimizer and trained using the same setup as the RunicNet.

The fully connected CNN with 8 layers base consists of several dense layers, starting with a Flatten layer to convert the input images into a one-dimensional array. The network had multiple FC layers, with 1024, 512, 256, 128, 64, 32, 16, and 8 neurons, each followed by the ReLU activation. Finally, the output layer uses a softmax activation function, producing probabilities for the classification task across the 4 classes. The total number of trainable parameters was 118.72 M.

### Evaluation Metrics

We evaluated the model’s performance on the testing dataset, which consists of 3 categories: healthy, unhealthy, and rubbish. The primary evaluation metric was the F1-score, calculated individually for each class and then weighted-average to produce an overall score. The F1-score was chosen as the primary metric for its balanced evaluation of precision and recall, crucial in medical diagnoses where both false positives and negatives can have serious consequences. Furthermore, it is a suitable metric for datasets with potential class imbalance. In addition to the F1-score, confusion matrices were generated to further assess the model’s performance. The confusion matrix examines the classification outcomes by categorizing results into true positives (TP), true negatives (TN), false positives (FP), and false negatives (FN). From the confusion matrix results, we computed precision (equation (2)), which measures how many of the predicted positive instances are correct; recall (equation (3)), which measures how well the model captures the actual positive instances; and F1-score (equation (4)), a balanced measure of precision and recall. These metrics are derived from the confusion matrix using the following definitions:

(2) $$Precision=\frac{TP}{TP+FP}$$

(3) $$Recall=\frac{TP}{TP+FN}$$

(4) $$F1\;score=2\times \frac{Precision\times Recall}{Precision+Recall}$$

## Results and Discussion

**Figure 3.**
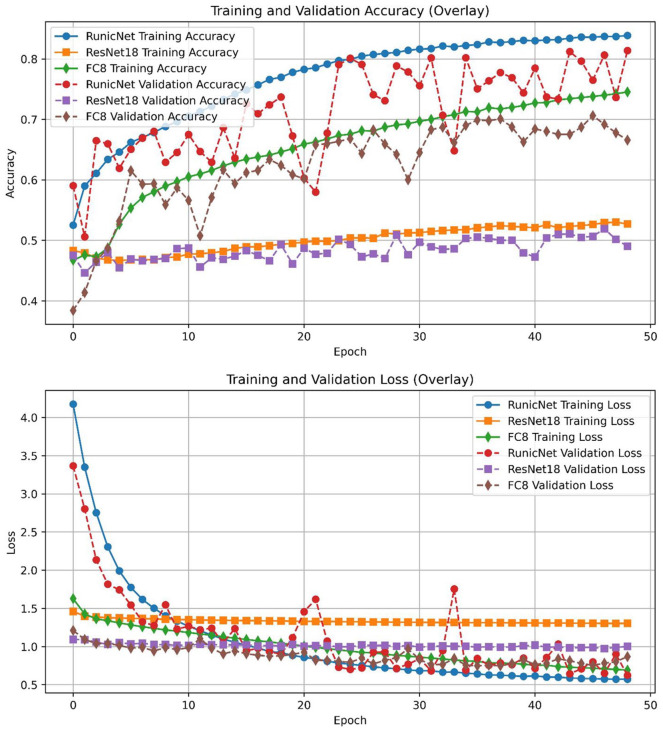
Training and validation accuracy and loss. The top panel illustrates the training and validation accuracy of three different models during training. The bottom panel shows the corresponding loss evolution during training over 49 epochs.

RunicNet demonstrated superior performance on the test dataset, achieving an F1-score of 0.78. In comparison, the ResNet-18 and FC CNN models yielded F1-scores of 0.53 and 0.66, respectively. These results highlight the effectiveness of our proposed architecture for cervical cancer classification. During training, RunicNet achieved an accuracy of 0.81, a loss of 0.62, a precision of 0.83, and a recall of 0.80 on the validation set. Figure 3 illustrates the overlaid loss and accuracy of the models during training. The performance of RunicNet can be attributed to its architectural components. The incorporation of the PA block, which includes re-parameterization, has been shown to be effective in high-level image-processing tasks^23,24^ and likely contributed to improved feature extraction. For high-dimensional data, convolutional filters may develop specific feature maps that fit the training data too closely, leading to poor generalization on the test dataset. L2 Regularization can help constrain weight magnitudes, reducing sensitivity to noise and improving robustness.^31,32^

To further account for possible overfitting, we applied batch normalization after each convolutional layer and incorporated dropout in the final layer. Furthermore, HFAB and the efficient attention mechanism exhibited stable performance with high reproducibility.

**Figure 4.**
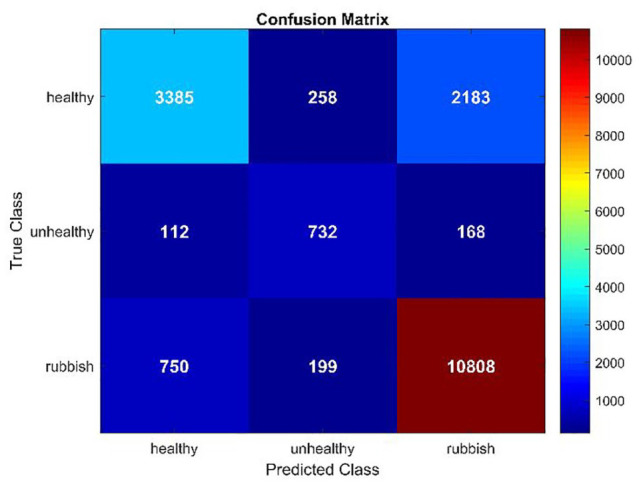
Confusion matrix of RunicNet on the test dataset.

As shown in the confusion matrix in Figure 4, whereas RunicNet correctly classified 3385 healthy, 750 unhealthy, and 10 808 rubbish images out of a total of 18 595 test images, there were notable misclassifications. Specifically, 2183 healthy images were incorrectly classified as rubbish, and 750 rubbish images were misclassified as healthy. The model performs best in detecting the “rubbish” category, with a high F1-score of 0.86, meaning that most rubbish samples were correctly classified. Conversely, the model struggled to identify the “unhealthy” category, achieving an F1-score of 0.65.

The lower F1-score of 0.65 for the “unhealthy” category is likely due to the imbalance in the dataset, where this class had the fewest training examples. Although we implemented strategies such as applying class weights to favor the “unhealthy” category and using additional data augmentation for this class during training, these efforts appear to have had a limited impact in fully compensating for the data disparity. Other factors beyond class imbalance may contribute to lower performance in the “unhealthy” class, particularly the challenge of capturing subtle morphological cues in low-resolution and blurry images. Future studies should consider alternative strategies for addressing class imbalance and incoherent image quality within the classes more effectively, such as exploring different data augmentation techniques, using synthetic data generation methods.^33^ Another promising avenue for future work involves investigating the use of alternative loss functions, such as focal loss,^34^ which tackles class imbalance by assigning more weight to difficult examples and less to easy ones. Our results emphasize the importance of dataset quality and distribution awareness when developing models for clinical use.

RunicNet achieved an overall weighted F1-score of 0.78 on the test dataset, demonstrating its capability in classifying the 3 categories: healthy, unhealthy, and rubbish. Through careful optimization of the learning rate, regularization (L2 with a weight decay of 0.001), and class weighting, we aimed to balance the learning process across both majority and minority classes. Even though these efforts enhanced model robustness, mitigated bias, and improved prediction reliability for underrepresented classes, further improvements are still needed.

There are several limitations to this study, including the use of a single-source test dataset to evaluate model performance. Although this dataset is a common benchmark, using data from multiple sources or different populations could provide a more robust assessment of the model’s generalizability to diverse real-world scenarios. Additionally, the study was retrospective and relied on cervical cell images that were already validated by experts. This means the model was trained and tested on a curated dataset, so its performance may vary when applied to raw, unvetted Pap smear images in a clinical setting. Also, while the proposed AI-based solution may provide an initial assessment or first opinion, potentially accelerating the reporting of Pap smear screenings, the pre-processing of images^10,21^ was substantial, involving advanced segmentation algorithms and background artifact removal. Ideally, image pre-processing should be automated to provide a truly end-to-end solution. Finally, further refinements to the model structure may yield additional performance improvements in future iterations.

## Conclusion

This study demonstrates the potential of advanced algorithmic solutions, particularly our proposed RunicNet model, in enhancing automated cytology. RunicNet achieved a promising weighted F1-score of 0.78 on the test dataset, surpassing traditional CNN architectures like ResNet-18 and an 8-layer fully connected CNN. This advancement holds promise for improving disease management, especially in underserved regions with limited access to expert pathologists, helping address global healthcare disparities.
